# Variants of *SCARB1* and *VDR* Involved in Complex Genetic Interactions May Be Implicated in the Genetic Susceptibility to Clear Cell Renal Cell Carcinoma

**DOI:** 10.1155/2015/860405

**Published:** 2015-04-06

**Authors:** Ewelina Pośpiech, Janusz Ligęza, Wacław Wilk, Aniela Gołas, Janusz Jaszczyński, Andrzej Stelmach, Janusz Ryś, Aleksandra Blecharczyk, Anna Wojas-Pelc, Jolanta Jura, Wojciech Branicki

**Affiliations:** ^1^Institute of Zoology, Faculty of Biology and Earth Sciences, Jagiellonian University, Gronostajowa 9, 30-387 Cracow, Poland; ^2^Faculty of Biochemistry, Biophysics and Biotechnology, Jagiellonian University, Gronostajowa 7, 30-387 Cracow, Poland; ^3^Centre of Oncology, Maria Skłodowska-Curie Memorial Institute, Garncarska 11, 31-115 Cracow, Poland; ^4^Department of Dermatology, Collegium Medicum of the Jagiellonian University, Skawińska 8, 31-066 Cracow, Poland

## Abstract

The current data are still inconclusive in terms of a genetic component involved in the susceptibility to renal cell carcinoma. Our aim was to evaluate 40 selected candidate polymorphisms for potential association with clear cell renal cell carcinoma (ccRCC) based on independent group of 167 patients and 200 healthy controls. The obtained data were searched for independent effects of particular polymorphisms as well as haplotypes and genetic interactions. Association testing implied position rs4765623 in the *SCARB1* gene (OR = 1.688, 95% CI: 1.104–2.582, *P* = 0.016) and a haplotype in *VDR* comprising positions rs739837, rs731236, rs7975232, and rs1544410 (*P* = 0.012) to be the risk factors in the studied population. The study detected several epistatic effects contributing to the genetic susceptibility to ccRCC. Variation in *GNAS1* was implicated in a strong synergistic interaction with *BIRC5*. This effect was part of a model suggested by multifactor dimensionality reduction method including also a synergy between *GNAS1* and *SCARB1* (*P* = 0.036). Significance of *GNAS1-SCARB1* interaction was further confirmed by logistic regression (*P* = 0.041), which also indicated involvement of *SCARB1* in additional interaction with *EPAS1* (*P* = 0.008) as well as revealing interactions between *GNAS1* and *EPAS1* (*P* = 0.016), *GNAS1* and *MC1R* (*P* = 0.031), *GNAS1* and *VDR* (*P* = 0.032), and *MC1R* and *VDR* (*P* = 0.035).

## 1. Introduction

Kidney cancer is according to the International Agency for Research on Cancer the third fastest increasing carcinoma with the 30% growth noted in years 1993–2009. Clear cell renal cell carcinoma (ccRCC) is the most often type of kidney cancer in humans [1, 2]. Several factors including obesity, hypertension, and smoking have been shown to increase risk of developing renal cell carcinoma [3, 4]. It has also been noted that this cancer is twice more frequent in males than in females [5]. So far only few genes have been associated with increased risk of RCC. The data obtained from three genome wide association (GWA) studies performed on European ancestry populations are inconsistent. First GWAS revealed three loci located on 2p21, 11q13.3, and 12q24.31 to be associated with RCC [6]. The gene* EPAS1* located in the region 2p21 has been previously suggested as a candidate gene in RCC due to its overexpression in this carcinoma [7]. The region 12q24.31 contains the* SCARB1* gene that encodes receptor involved in uptake of high-density lipoprotein cholesterol. Fine mapping analysis of a 120 kb area near* EPAS1* has confirmed association of 2p21 region with RCC but stronger signal was noted outside* EPAS1* [8]. The second GWAS has implicated the* ITPR2* gene on 12p.11.23 as a novel susceptibility locus for RCC while no other loci reached genome-wide significance in this extended population sample [9]. The third GWA study identified additional association with RCC for* ZEB2* gene encoding zinc finger E-box-binding homeobox 2 [10]. The recent GWAS conducted on samples from African Americans has revealed the region 11q13.3 containing no genes to be associated with RCC, confirming the outcome obtained before for Europeans [6, 11]. Other loci implicated in RCC include* GNAS1* showing a prognostic value [12] and several genes which have been associated with RCC risk. These latter ones include two genes from the vitamin D pathway, namely,* VDR* and* RXRA* which encode proteins forming a complex involved in vitamin D dependent transcription regulation [13] and polymorphism in the promoter of the* VEGFA* gene [14]. Two studies have reported associations with RCC in Chinese population showing polymorphism in the promoter of the* BIRC5* gene [15] and variation in the* XRCC6* locus, to be the susceptibility candidates for renal cell carcinoma [16]. One study indicated insertion/deletion polymorphism in the promoter region of* CASP8* encoding a key regulator of apoptosis, caspase-8, to be a factor in RCC susceptibility and metastasis [17]. To further investigate the problem of genetic susceptibility to RCC we analysed 35 candidate polymorphisms within genes previously implicated in RCC development and prognosis. Additionally, 5 SNPs located in novel gene* ZC3H12A* encoding MCP-1-induced protein (MCPIP1) involved in a regulation of inflammatory reaction were included in the analyses. MCPIP1 acts as an RNAse regulating stability of some mRNA coding for proinflammatory cytokines and also regulates activity of transcription factors, which are key regulators in carcinogenesis (e.g., NF-*κ*B) [18–21]. Therefore, we hypothesize that variation in* ZC3H12A *may be associated with the risk of ccRCC development. Performed analyses included examination of main effects associated with all the selected 40 polymorphisms and the reconstructed haplotypes in* EPAS1*,* RXRA*, and* VDR* as well as a thorough search for epistatic effects using multifactor dimensionality reduction (MDR) and logistic regression methods.

## 2. Materials and Methods

### 2.1. Study Population and Candidate Genetic Loci

This research was approved by the bioethical commission of the Regional Research Council in Kraków, number 68/KBL/OIL/2011. All the subjects gave an informed consent. The study sample comprised 167 ccRCC patients treated with a surgery in the Centre of Oncology in Kraków and 200 healthy individuals frequency matched by sex and age. Tissues collected from ccRCC patients were subjected to DNA extraction using silica-based method with GeneJET Genomic DNA Purification Kit (Thermo Fisher Scientific Inc., Waltham, MA) using the manufacturer's protocol. DNA from the buccal swabs collected from the control group was extracted by silica adsorption using the NucleoSpin Tissue extraction kit (Macherey-Nagel GmbH & Co. KG, Germany) as previously described [22]. Forty candidate polymorphisms including eight SNPs previously associated with renal cell carcinoma through the 4 genome wide association studies [6, 9–11] and seven SNPs associated with RCC in the selected paramount candidate gene approach association studies [12–17] were included in the research. Additionally, nine most often investigated SNPs in the* VDR* gene were examined due to a suggested association of this locus with RCC [13]. Eleven most relevant SNPs in* MC1R*, which is the important melanoma associated gene, were included due to a postulated increased risk of RCC in melanoma patients [2]. The remaining 5 SNPs are located in the* ZC3H12A* gene, which is involved in regulation of inflammatory reaction [20]. Details on the selected forty polymorphisms are given in Table 1.

### 2.2. Genotyping

Polymorphisms were genotyped in 5 multiplex and 1 singleplex PCR reactions. SNPs in multiplexes were amplified using Qiagen Multiplex PCR kit (Qiagen, Hilden, Germany) and further analysed with single base extension (SBE) reactions using SNaPshot Multiplex Kit (Applied Biosystems, Foster City, CA). Multiplex 1 included 7 SNP positions (rs11894252, rs7579899, rs833061, rs3118523, rs748964, rs7105934, and rs4765623), multiplex 2 consisted of 6 SNPs (rs9679290, rs4953346, rs1049380, rs9904341, rs7121, and rs132770), and multiplex 3 comprised further 6 SNPs (rs34796867, rs113322875, rs34031609, rs113655247, rs17849897, and rs12105918). Sequences and concentrations of PCR and SBE primers are shown in Supplementary Tables  1 and  2, respectively in Supplementary Material available online at http://dx.doi.org/10.1155/2015/860405. Amplifications were performed in 5 *μ*L reaction volume consisting of 2.5 *μ*L Qiagen Multiplex PCR mixture, 0.5 *μ*L of Q solution, 0.5 *μ*L of primer premix, and 1.5 *μ*L of template DNA. The PCR reactions were carried out with the following temperature profile: 95°C/15 min, [94°/30 s, 58°C/90 s, 72°C/90 s] × 32, 72°C/10 min. The amplification products were cleaned with a mixture (1 : 1) of Exonuclease I (Exo I) and FastAP Thermosensitive Alkaline Phosphatase (FastAP) enzymes (Thermo Fisher Scientific Inc., Waltham, MA). The minisequencing reactions consisted of 0.5 *μ*L of SNaPshot mix, 0.5 *μ*L of SBE primer premix, 1 *μ*L of purified PCR product, and 3 *μ*L of DNase free water. The SBE temperature profile was as follows: [96°C/10 s, 50°C/5 s, 60°C/30 s] × 26. Products of the extension reactions were purified with 1 *μ*L of FastAP Thermosensitive Alkaline Phosphatase enzyme and analyzed with ABI 3100 Avant Genetic Analyser (Applied Biosystems, Foster City, CA). The remaining 11 polymorphisms in* MC1R* (N29insA, rs1805005, rs1805006, rs2228479, rs11547464, rs1805007, Y152OCH, rs1110400, rs1805008, rs885479, and rs1805009) and 9 SNPs from the* VDR* gene (rs739837, rs731236, rs7975232, rs1544410, rs2228570, rs2238136, rs4516035, rs7139166, and rs11568820) were analysed in additional two multiplexes using protocols described elsewhere [22, 23]. The remaining insertion/deletion polymorphism in* CASP8* was analysed in a single PCR reaction using HotStarTaq Master Mix Kit (Qiagen, Hilden, Germany) and following primer sequences 5′-6-FAM-AAACTTCTCCCATGGCCTCT-3′ and 5′-TATGAATGAGCCGAGGAAGG3′. Amplifications were carried out in 5 *μ*L reaction volume consisting of 2.5 *μ*L HotStarTaq Master Mix, 0.5 *μ*L of primer premix (concentration of 0.125 *μ*M), 0.5 *μ*L of distilled water, and 1.5 *μ*L of template DNA. The following temperature profile was applied: 95°C/15 min, [94°/30 s, 60°C/45 s, 72°C/60 s] × 30, 72°C/10 min. PCR products were directly analyzed by capillary electrophoresis using ABI 3100 Avant Genetic Analyser (Applied Biosystems, Foster City, CA).

### 2.3. Statistical Analyses

#### 2.3.1. Population Analyses and Haplotype Reconstruction

Genetic data obtained for the 40 selected polymorphisms were tested for deviations from Hardy-Weinberg equilibrium. Hardy-Weinberg analysis was performed with Arlequin version 3.1 software (http://cmpg.unibe.ch/software/arlequin3/). Haploview version 4.2 software was used to test the degree of linkage disequilibrium among DNA variants in* EPAS1*,* RXRA*, and* VDR* (http://www.broadinstitute.org/). Haplotype reconstruction and frequency estimation were done for SNPs located in* EPAS1*,* RXRA*, and* VDR* using PHASE version 2.1 computer software (http://stephenslab.uchicago.edu/software.html#phase). Haplotype reconstruction and LD analysis did not include polymorphisms in the* MC1R* gene because they all may individually affect receptor performance [24] and polymorphisms in* ZC3H12a* as besides one SNP (rs17849897) they turned out to be monomorphic in the tested population. In the case of* VDR* gene, polymorphisms were divided into 3 blocks following the obtained results and literature suggestions [25, 26]. Positions rs739837, rs731236, rs7975232, and rs1544410 were included in the first block. The second block was formed by a single rs2228570 position whereas in the third block the remaining rs2238136, rs4516035, rs713966, and rs11568820 positions were considered. Minimal ORs detectable with the power of at least 80% were calculated with Power and Sample Size Program (PS Program) version 3.1.2 (http://biostat.mc.vanderbilt.edu/wiki/Main/PowerSampleSize).

#### 2.3.2. Association Testing

Univariate associations between single selected polymorphisms and ccRCC status were assessed through binary logistic regression with PASW statistics version 21 computer software (SPSS Inc., Chicago, IL, USA). Odds ratios (ORs) with corresponding 95% confidence intervals (CIs) and *P* values were assigned. Additive, recessive, and dominant models of allele categorization were tested. In case of the* MC1R* gene, polymorphisms were divided in two groups according to their known influence on pigmentation phenotype. Strong variants marked with “*R*” (N29insA, rs1805006, rs1805007, rs11547464, rs1805008, rs1805009, and Y152OCH) have been considered as high-penetrance since they significantly diminish the receptor performance and have strong phenotypic effect (OR values of ~50 or higher). Weak variants marked with “*r*” are low-penetrance and have weaker effect on the receptor performance and therefore weaker phenotypic effect (OR below ~10). Such a categorization has been applied in several previous studies (e.g., [27]). For “*R*” variants, three states were considered according to the existence of major function mutations (0 = no “*R*” variant carriers, 1 = one “*R*” variant carriers, and 2 = two “*R*” variant carriers) and the same approach was used in case of “*r*” variants. Binary logistic regression was also used to evaluate the association of the inferred haplotypes (with frequency exceeding 0.5%) under the assumption of additive inheritance mode. Association analyses were conducted considering *P* value of <0.05 level as statistically significant; however associations were also tested with false discovery rate (FDR) correction for multiple comparisons (http://www.seu.ac.lk/cedpl/student download/BenjaminiHochberg.xlsx). Finally, multivariate logistic regression was applied including simultaneous analysis of all the polymorphisms and haplotypes as well as disclosed interactions.

#### 2.3.3. Epistasis Examination

Statistical locus-locus interactions were examined using two methods, that is, multifactor dimensionality reduction (MDR v2.0 beta 8.1 http://www.epistasis.org/) and binary logistic regression (PASW statistics v.21). MDR approach enables evaluation of epistatic effects through assignment of all genotype combinations between interacting loci to one of the two groups: “high-risk” or “low-risk” of disease development based on the ratio of cases to controls and the analysis is performed as described elsewhere [28]. The results of MDR analysis were interpreted using entropy-based approach which is a nonparametric method for estimation of the benefit in information gain from interaction between two analyzed attributes [29]. In addition to MDR analysis, the parametric binary logistic regression method was applied. Pairwise interactions were tested by introducing interaction terms into the two-factor logistic regression models using forward selection strategy. ORs with 95% CIs and respective *P* values were estimated for interactions disclosed with logistic regression.

#### 2.3.4. Meta-Analysis

Meta-analysis for rs4765623 in* SCARB1* was performed with MetaEasy v1.0.5 [30] and involved data from our population and data for three different sample sets (IARC, NCI, Replication) from the study of Purdue et al. (2011) [6]. OR value for the combined data was calculated with the fixed effects model and *P* value was estimated based on 95% CI with the method described in Altman and Bland [31]. Heterogeneity was investigated using Cochrane Q *P* value and *I* ^2^ statistics which assessed the consistency in meta-analysis studies. *I* ^2^ ranges from 0% to 100% where 0% indicates no heterogeneity whereas increasing value of *I* ^2^ indicates increasing level of heterogeneity.

## 3. Results

### 3.1. Population Analyses and Haplotype Inference

No significant departures from the Hardy-Weinberg equilibrium were noted after Bonferroni's correction for multiple testing (*P* > 0.0013) for the examined loci. Results of LD analysis among DNA variants investigated in* EPAS1*,* RXRA*, and* VDR *are presented in Supplementary Figure  1. Two haplotype blocks (rs11894252-rs7579899; rs9679290-rs4953346) with strong LD (*r* ^2^ > 0.9) were found in the* EPAS1* gene, moderate linkage disequilibrium was revealed for two SNPs rs748964-rs3118523 (*r* ^2^ = 0.65) in the* RXRA* gene whereas in the* VDR* gene strong linkage for two SNPs rs4516035-rs7139166 (*r* ^2^ > 0.9) and 4 SNPs-haplotype block comprising rs739837-rs731236-rs7975232-rs1544410 (*r* ^2^ > 0.5) were detected. From 7 haplotypes reconstructed with PHASE program for all SNPs in* EPAS1*, 4 were very frequent. In case of* RXRA* 3 frequent and 1 rare haplotypes were reconstructed. In the block number 1 in* VDR*, comprising 4 SNPs, 10 haplotypes were reconstructed but only 6 were found to have frequency above 0.5%. In case of block number 3 in* VDR* 11 haplotypes were reconstructed with 7 exceeding the 0.5% threshold. The reconstructed haplotypes and their frequencies are listed in Supplementary Table  3.

### 3.2. Association and Interaction Analyses

Samples collected from patients and controls were frequency matched by sex (58.1% of males in the ccRCC group and 51% in the control group) and age (the mean age of the participants was 64 years in the ccRCC group and 62 years in the control group). Univariate analyses performed for the particular 40 polymorphisms under study revealed rs4765623 in the* SCARB1* gene to be associated with ccRCC in our population but only considering dominant mode of inheritance (*P* = 0.016) and this result did not pass FDR procedure. According to logistic regression analysis individuals with at least one minor A allele have 1.7 higher odd of ccRCC development comparing to individuals with GG genotype (OR = 1.7, 95% CI: 1.104–2.582). Additive mode of allele inheritance did not reach the threshold of 0.05 significance (*P* = 0.075). Except for rs4765623 in* SCARB1* no other SNP showed main effect in our study. We also could not verify significance of 4 SNPs in the* ZC3H12a* gene which were found to be monomorphic in this sample set. The only polymorphic position in* ZC3H12a* (rs17849897) with the minor allele frequency of 1.4% turned out not to be associated with ccRCC. Results of univariate association analyses are provided in Supplementary Table 4.

An association testing performed for the reconstructed haplotypes revealed that CAGT haplotype in* VDR* gene (comprising rs739837-rs731236-rs7975232-rs1544410) observed with frequency 0.68% might be a risk factor for ccRCC (*P* = 0.012). This result was also found to be insignificant after applying the FDR procedure. Notably, however, CAGT haplotype was observed in 5 among 167 cases of ccRCC but was completely absent in the control group of 200 individuals. Haplotypes reconstructed in the* EPAS1* and* RXRA* did not reveal any association with ccRCC under the significance threshold of 0.05.

**Figure 1:**
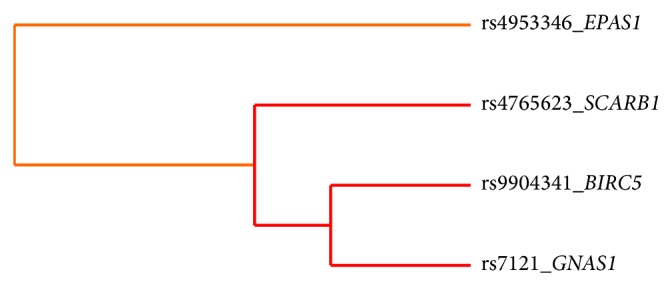
Interaction dendrogram provided with MDR analysis. Dendrogram interaction graphs are built using hierarchical cluster analysis and illustrate the presence, strength, and nature of epistatic effects. The more the line connecting two factors is moved to the right side, the stronger the interaction effect is. The red line between factors indicates a high degree of synergy whereas the orange line indicates a lesser degree of synergy.

**Figure 2:**
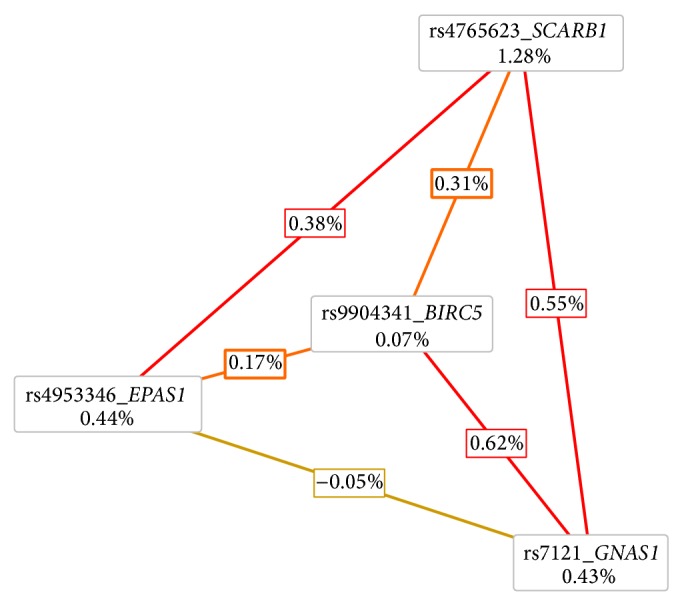
Entropy-based interaction radial graph provided with MDR analysis. Entropy values in the cells of individual factors indicate the main independent effects whereas the entropy values marked on the lines connecting two factors represent the effect of interaction.

The study revealed several epistatic effects involving the abovementioned DNA variants in* SCARB1* and* VDR*, which confirmed suggestive association trends with ccRCC. According to MDR analysis, ccRCC is best explained by the four-factor model comprising rs4765623 in* SCARB1*, rs4953346 in* EPAS1*, rs9904341 in* BIRC5*, and rs7121 in* GNAS1* with balanced accuracy (BA) of 0.5828, cross-validation consistency (CVC) of maximum level 10/10, and permutation testing *P* value of 0.036 (Table 2). Analysis of interaction dendrogram provided by MDR confirmed implication of these factors in epistatic effects showing strong positive (synergistic) interactions between rs7121 in* GNAS1*, rs9904341 in* BIRC5*, and rs4765623 in* SCARB1* (Figure 1). Entropy-based interaction graph confirmed strong positive effects between rs7121 and rs9904341 and between rs7121 and rs4765623. According to the entropy-based analysis the largest main effect is attributed to rs4765623 in* SCARB1*. This position removes 1.28% of entropy, what is understood to remove 1.28% of “uncertainty” in prediction of ccRCC status. The position rs7121 in* GNAS1* eliminates 0.43% of entropy, whereas the interaction between these two factors removes additional 0.55% of entropy which is not removed by any of these factors treated individually. In case of the second interaction revealed, between rs7121 in* GNAS1* and rs9904341 in* BIRC5* positive value of entropy brought by interaction equaled 0.62% which indicates additional benefit in information gain when considering epistasis between these factors (Figure 2).

Pairwise interaction testing performed with logistic regression confirmed significance of rs4765623-rs7121 interaction (*P* = 0.041) and revealed 5 additional epistatic effects (Table 3). The highest statistical significance was noted for interaction between rs4765623 in* SCARB1* and rs9679290 in* EPAS1* (*P* = 0.008). Statistical significance was also noted for interaction between rs7121 in* GNAS1* and rs9679290 in* EPAS1* (*P* = 0.016). The remaining interactions of potential influence on ccRCC include* GNAS1*–*MC1R* (*P* = 0.031),* GNAS1*–*VDR* (*P* = 0.032), and* MC1R*-*VDR* (*P* = 0.035). Overall, 6 genes (*SCARB1*,* GNAS1*,* EPAS1*,* BIRC5*,* MC1R*, and* VDR*) were found to be implicated in interaction effects of potential influence on ccRCC development (Table 3).

Finally, multivariate logistic regression involving simultaneous analysis of all the studied polymorphisms, haplotypes, and gene-gene interactions revealed the following factors to be relevant in susceptibility to ccRCC: CAGT haplotype in* VDR* (*P* = 0.005), interaction between rs4765623 in* SCARB1* and rs9679290 in* EPAS1* (*P* = 0.007), interaction between variants “*r*” in* MC1R* and rs7975232 in* VDR* (*P* = 0.010), and interaction between rs7121 in* GNAS1* and rs2228570 in* VDR* (*P* = 0.041) (Table 4). This final logistic regression model has the significance of *χ* ^2^ *P* = 6.98 × 10^−6^ and explains 9.2% of the total risk in ccRCC development.

### 3.3. Results of Meta-Analysis

Meta-analysis of the pooled data from Purdue et al. [6] and the results of our study for rs4765623 in* SCARB1* indicated that moderate heterogeneity may be present among the tested population samples with *I* ^2^ = 62.10% and Cochrane Q  *P* value = 0.048. Effect size of rs4765623 in* SCARB1* calculated for the combined data was OR = 1.145, 95% CI: 1.093–1.200 with *P* = 2.483 × 10^−8^.

## 4. Discussion

The picture arising from the available genetic data for renal cell carcinoma is ambiguous with no single polymorphism showing a record of repeatable and reliable association with RCC in independent studies. Here, we analyzed a group of 167 ccRCC patients and 200 healthy controls examining 40 candidate polymorphisms in 14 loci selected from the literature [2, 6, 9, 10, 12–19].

The study revealed rs4765623 in the* SCARB1* gene to show association trend with ccRCC in the studied population sample. The* SCARB1* gene encodes the scavenger receptor class B member 1 which binds to the high-density lipoprotein (HDL) cholesterol and therefore mediates cellular cholesterol homeostasis. Cholesterol transfer towards liver for excretion is known as a protective mechanism against the development of atherosclerosis [32]. The role of* SCARB1* in cancer biology is not well investigated but it has been recently suggested that SCARB1 receptor may be involved in multiple functions [33] and that cholesterol entry through SCARB1-HDL can activate signal transduction pathways that can regulate cellular proliferation and migration and therefore carcinogenesis [34]. Association of rs4765623 position in* SCARB1* with RCC was first identified in GWAS study performed on European population by Purdue et al. in 2011 [6]. The revealed effect was weak (OR = 1.15) but significant (*P* = 2.6 × 10^−8^). However, this association has not been confirmed in replication studies performed on Chinese population [35, 36]. Our results confirmed association of the rs4765623 minor allele A (*f* = 36.1%) with susceptibility to ccRCC in European (Polish) population sample and can serve as an independent replication to further establish the association for rs4765623 for renal cell carcinoma risk. The effect identified in our study (OR = 1.7) was stronger than in the study of Purdue but this value was observed when dominant mode of minor allele classification was applied. Frequency of A risk allele reported in Purdue et al. was similar to our study (34%). Purdue et al. has reported significant heterogeneity for rs4765623 in* SCARB1* (*P* = 0.03) among the three studied groups [6]. Meta-analysis including our data also suggests moderate interpopulation heterogeneity with ~62% *I* ^2^ value but the significance decreased from 0.03 to 0.048. The effect size of rs4765623 in* SCARB1* for the combined data, including our population, was very similar to that obtained in Purdue et al. (OR = 1.145, *P* = 2.483 × 10^−8^).

Our study also revealed suggestive association trend of* VDR* haplotype comprised of four SNPs, namely, rs739837, rs731236, rs7975232, and rs1544410, with ccRCC risk.* VDR* gene encodes vitamin D receptor which is involved in vitamin D turnover. The active form of vitamin D [1,25(OH)_2_D_3_] is considered to be a protective factor against cancer development due to its role in cell growth regulation, differentiation, programmed cell death, angiogenesis, and moderation of gene transcription [37, 38]. Indeed, several functional studies have shown that the level of the active form of vitamin D is significantly lower in ccRCC patients than in healthy controls [39, 40]. The active form of vitamin D binds to the vitamin D receptor which is predicted to regulate expression of over 2000 genes in humans [41]. Notably, variation in* VDR* gene has been implicated in many carcinomas (e.g., [13, 42–47]). However, few reports are available on association between* VDR* polymorphism and renal cell carcinoma and the obtained results are inconsistent [48, 49]. Most of the previous studies have been performed on Asian populations [50, 51] and recent meta-analyses showed that association of* VDR* polymorphism with ccRCC may be population specific [52, 53]. Polymorphisms included in the haplotype associated with ccRCC in our study are located in the 3′ region of* VDR* gene and are supposed to influence mRNA level [54]. Allele T of rs731236 (*TaqI*) polymorphism was associated with increased risk of RCC in a Japanese population sample [50] and then confirmed in a population of European descent [13]. However, several other studies have not confirmed that association (e.g., [51]). Our research revealed increased risk of RCC development associated with allele T in rs731236 when considered in the haplotype with three other SNPs. Interestingly, the identified risk haplotype CAGT was absent in a group of 200 healthy controls. Moreover, we analysed* VDR* variation in additional 300 controls and did not observe this haplotype (*P* = 0.008, data not shown). Association of a haplotype in the* VDR* gene with RCC was also noted by Karami et al. but in that study different region of the* VDR* gene was involved [13].

The discovered main association effects need to be interpreted as trends only because they did not pass FDR procedure. Small number of samples was an obvious limitation of this study that could prevent detection of weak genetic effects known to be typical for ccRCC. However, nonreplication may have also been caused by interpopulation heterogeneity [55] which was observed in meta-analysis for rs4765623 in* SCARB1*. Our sample size was sufficient to detect the true effects of OR = 1.803–1.941 (depending on minor allele frequency) with 80% probability for 19 out of 40 tested DNA variants. Among variants associated with RCC through GWA studies the strongest effect has been shown so far for rs7105934 (11q13.3) with OR equaling 1.41 in European samples [6] and 1.79 in African American samples [11]. It seems therefore that most of the known genetic RCC risk factors might have escaped detection in this study. However, besides main effects of the studied loci we also used statistical methods to test interactions between them. Notably, the* SCARB1* locus was implicated by the MDR method in complex gene-gene interactions in the studied population sample. The significance of epistasis in the determination of complex traits is currently often emphasized and it has been suggested that the impact of genetic interactions may even outrank an independent main effect of single susceptibility loci [56]. Importantly, the applied MDR approach was developed as nonparametric and genetic model-free data mining strategy for epistasis identification in studies dealing with relatively small sample size [28, 57]. It has been postulated that this method is less prone to I type error and thus more reliable than logistic regression. MDR showed that four-factor model comprising two synergistic interactions, the first between rs7121 in* GNAS1* and rs9904341 in* BIRC5* and the second between rs7121 in* GNAS1* and rs4765623 in* SCARB1*, best explains susceptibility to ccRCC. The* GNAS1* gene encodes a *α*s subunit of heterotrimeric G protein that is required for activation of adenylyl cyclase and generation of cAMP and plays the key role in multiple signal transduction pathways, linked to proapoptotic processes in cancer cells. Modulated expression of this gene has been associated with various disorders (e.g., [58, 59]).* GNAS1* has been linked to RCC in two independent studies [12, 60]. The* BIRC5* gene encodes survivin, a protein preventing apoptotic cell death. Overexpression of this gene has a continuous literature record of its prognostic significance in many carcinomas (e.g., [61, 62]). Increased expression of survivin has also been reported in RCC [63]. Recently, promoter mutation rs9904341 in* BIRC5* has been associated with susceptibility to RCC in Asians [15]. There is evidence that this polymorphism regulates transcriptional activity increasing survivin level [64]. Notably, there are evidences that there is a direct dependency between* GNAS1* and* BIRC5* products. Subunit *α*s activates STAT3 transcription factor inducing signal through the PKA kinase, JNK, and PI3 K [65]. In turn, STAT3 is important in activation of survivin encoded by* BIRC5* gene (e.g., [66]). Therefore, synergistic interaction of* GNAS1* and* BIRC5* in susceptibility to ccRCC discovered in this study seems to be justified. In one of the recent studies utilizing ccRCC data provided by the Cancer Genome Atlas (TCGA), another member of the same group of inhibitors of apoptosis* BIRC7* has been listed among genes differentially expressed in ccRCC [67]. The MDR method also revealed that rs7121 in* GNAS1* is also implicated in synergistic interaction with rs4765623 in* SCARB1*. This interaction was further confirmed by logistic regression (*P* = 0.041). Interestingly, logistic regression also revealed additional and more statistically significant epistatic effect with* SCARB1* as a component, that is, interaction between rs4765623 in* SCARB1* and rs9679290 in* EPAS1* (*P* = 0.008). Although a direct dependency between* EPAS1* and* SCARB1* or their protein products is unclear, it is worth noting that both these loci are related to the processes connected with angiogenesis. The* SCARB1* gene, as it was mentioned before encodes receptor that binds to the high-density lipoprotein (HDL). It has been suggested that beyond its best known function in cholesterol mediation, HDL is also involved in many different activities like anti-inflammatory, antiapoptotic processes and a variety of endothelial behaviors and therefore angiogenesis (e.g., [68, 69]). The second locus involved in the revealed interaction is* EPAS1* which was associated with ccRCC in a large GWA study performed by Purdue et al. [11]. This gene encodes a hypoxia-inducible factor 2 (HIF2*α*) which belongs to the transcription factors responsible for induction of genes controlled by oxygen. Under normal conditions, the level of HIFs is regulated by ubiquitinase complex, comprising a von Hippel-Lindau tumor suppressor protein (pVHL). In normoxia HIF factors are ubiquitinated and degraded. With a drop of oxygen level (e.g., in tumors), stabilization of HIF1*α* and HIF2*α* (encoded by* EPAS1*) occurs, which is what results in induction of transcription of many genes encoding proteins involved in angiogenesis [70]. Inactivation of* VHL* gene is observed in approximately 90% of patients with clear cell RCC and leads to accumulation of HIF1*α* and HIF2*α* proteins and in consequence to stimulation of expression of protooncogenes* TGFα* and* c-Met* [7, 71, 72].

The* GNAS1* gene was found in our study to be implicated in other epistatic effects with* MC1R* (*P* = 0.031),* EPAS1* (*P* = 0.016), and* VDR* (*P* = 0.032). The* MC1R* gene encodes melanocortin 1 receptor which is involved in the regulation of melanin pigment synthesis which has also been found to increase the risk of developing melanoma (e.g., [44, 73, 74]). It is well established that patients with cancer are at higher risk to develop multiple cancers and some examples of melanoma and RCC coexistence have been reported (e.g., [75, 76]). However, risk factors common for both cancers are only partially explained and include mutations in* MITF *gene [77]. Interestingly, product of* MC1R* gene acts through G-protein. Therefore, there is a direct molecular dependence between* MC1R* and* GNAS1* products. Importantly, it has been demonstrated that* MC1R* is expressed in the kidney cells and shown that treatment with MC1R agonists ameliorated kidney diseases in rats with passive Heymann nephritis [78]. Finally, logistic regression also indicated interaction between the* MC1R* and* VDR* genes (*P* = 0.035). It is not clear how this epistatic effect could affect ccRCC development, but interaction between these two genes has been suggested to influence human pigmentation [23, 79]. Interestingly, in the recent TCGA study another gene involved in melanogenesis and increased risk of melanoma that is the* TYRP1* locus [74] has been selected as differentially expressed in ccRCC [67]. The overlap between risk factors in melanoma and ccRCC seems to be interesting and worth further investigation.

In conclusion, position rs4765623 in* SCARB1* and haplotype in* VDR* showed suggestive association trends with ccRCC susceptibility in the studied Polish population. Moreover using MDR and logistic regression methods, a complex network of interactions involving six genes previously implicated in RCC (*SCARB1*,* GNAS1*,* BIRC5*,* EPAS1*,* VDR*, and* MC1R*) was discovered. Due to a relatively small sample number these epistatic effects should be further studied and confirmed on a larger cohort. The risk haplotype in* VDR* and the gene-gene interactions* SCARB1*-*EPAS1*,* GNAS1*-*VDR*, and* MC1R*-*VDR* were included in the final logistic regression model explaining 9.2% of the total risk in ccRCC development.

## Supplementary Material

Supplementary Materials include four supplementary tables (PCR primer sequences for three multiplex reactions, Extension primer sequences for three multiplex reactions, Frequency of the haplotypes reconstructed with PHASE v2.1, Results of association logistic regression analysis between ccRCC status and single polymorphisms) and one supplementary figure (LD schemes generated with Haploview program for SNPs in EPAS1 (A), RXRA (B) and VDR (C) genes).

## Figures and Tables

**Table 1:** Data for the studied 40 polymorphisms.

Locus	Gene	Chromosome	Position (GRCh38)	Allele variants	MAF^∗^	Reference
rs34796867	*ZC3H12A *	1	37475773	C/T	T = 0.000	[20]
rs113322875	*ZC3H12A *	1	37477383	G/A	A = 0.000	[20]
rs34031609	*ZC3H12A *	1	37482928	G/A	A = 0.000	[20]
rs113655247	*ZC3H12A *	1	37483276	G/C	C = 0.000	[20]
rs17849897	*ZC3H12A *	1	37483451	C/T	T = 0.014	[20]
rs12105918	*ZEB2 *	2	144450626	G/A	G = 0.051	[10]
rs11894252	*EPAS1 *	2	46306237	C/T	T = 0.356	[6]
rs7579899	*EPAS1 *	2	46310465	C/T	T = 0.357	[6]
rs9679290	*EPAS1 *	2	46330505	G/C	C = 0.451	[6]
rs4953346	*EPAS1 *	2	46331069	C/A	C = 0.452	[6]
rs3834129	*CASP8 *	2	201232809:14	CTTACT/—	Del = 0.450	[17]
rs833061	*VEGFA *	6	43769749	C/T	C = 0.484	[14]
rs748964	*RXRA *	9	134442243	G/C	G = 0.144	[13]
rs3118523	*RXRA *	9	134443675	G/A	G = 0.202	[13]
rs7105934	11q13.3	11	69424973	C/T	T = 0.040	[6]
rs1049380	*ITPR2 *	12	26336611	G/T	G = 0.436	[9]
rs739837	*VDR *	12	47844438	C/A	A = 0.471	[13]
rs731236	*VDR *	12	47844974	G/A	G = 0.354	[13]
rs7975232	*VDR *	12	47845054	G/T	T = 0.469	[13]
rs1544410	*VDR *	12	47846052	C/T	T = 0.361	[13]
rs2228570	*VDR *	12	47879112	G/A	A = 0.456	[13]
rs2238136	*VDR *	12	47883930	G/A	A = 0.365	[13]
rs4516035	*VDR *	12	47906043	C/T	C = 0.458	[13]
rs7139166	*VDR *	12	47906551	G/C	G = 0.454	[13]
rs11568820	*VDR *	12	47908762	C/T	T = 0.128	[13]
rs4765623	*SCARB1 *	12	124836304	G/A	A = 0.361	[6]
N29insA	*MC1R *	16	89919345	C/A	A = 0.000	[2]
rs1805005	*MC1R *	16	89919436	G/T	T = 0.078	[2]
rs1805006	*MC1R *	16	89919510	C/A	A = 0.001	[2]
rs2228479	*MC1R *	16	89919532	G/A	A = 0.108	[2]
rs11547464	*MC1R *	16	89919683	G/A	A = 0.007	[2]
rs1805007	*MC1R *	16	89919709	C/T	T = 0.040	[2]
Y152OCH	*MC1R *	16	89919714	C/A	A = 0.000	[2]
rs1110400	*MC1R *	16	89919722	C/T	C = 0.026	[2]
rs1805008	*MC1R *	16	89919736	C/T	T = 0.068	[2]
rs885479	*MC1R *	16	89919746	C/T	T = 0.035	[2]
rs1805009	*MC1R *	16	89920138	G/C	C = 0.001	[2]
rs9904341	*BIRC5 *	17	78214286	G/C	C = 0.343	[15]
rs7121	*GNAS1 *	20	58903752	C/T	T = 0.465	[12]
rs132770	*XRCC6 *	22	41621260	G/A	A = 0.153	[16]

^∗^MAF = minor allele frequency.

**Table 2:** Results of multifactor dimensionality reduction analysis performed for ccRCC status.

Multifactor dimensionality reduction analysis			
Best candidate model (for up to five factor combinations)	BA^∗^	CVC^∗∗^	*P*-value^∗∗∗^
rs4765623	0.5469	10/10	0.244
rs4765623; rs4953346	0.5125	4/10	0.658
rs4765623; rs4953346; rs9904341	0.5319	5/10	0.432
**rs4765623; rs4953346; rs9904341; rs7121**	**0.5828**	**10/10**	**0.036**
rs4765623; rs4953346; rs9904341; rs7121; rs132770	0.5598	10/10	0.140

Best model is marked with bold.

^∗^BA: balanced accuracy.

^∗∗^CVC: cross-validation consistency.

^∗∗∗^Permutation testing *P* value.

**Table 3:** Interaction effects of statistical significance in ccRCC development revealed with binary logistic regression analysis.

Interacting loci	Genes	OR (95% CI) for interaction model	*P* value for interaction model
rs4765623^∗^rs9679290	*SCARB1* ^∗^ *EPAS1 *	1.371 (1.087–1.729)	**0.008**
rs7121^∗^rs9679290	*GNAS1* ^∗^ *EPAS1 *	1.284 (1.047–1.575)	**0.016**
rs7121^∗^rs1805008	*GNAS1* ^∗^ *MC1R*	2.601 (1.089–6.212)	**0.031**
rs7121^∗^rs2228570	*GNAS1* ^∗^ *VDR *	1.240 (1.018–1.510)	**0.032**
MC1R “r”^∗^rs7975232	*MC1R* ^∗^ *VDR *	1.341 (1.020–1.763)	**0.035**
rs7121^∗^rs4765623	*GNAS1* ^∗^ *SCARB1*	1.639 (1.020–2.639)	**0.041**

**Table 4:** Results of multivariate binary logistic regression analysis concerning all SNPs under study, haplotypes, and disclosed interactions.

Multivariate binary logistic regression analysis			
Variables in the model	Genes	OR (95% CI)	*P* value of variable in the model
Haplotype CAGT (rs739837-rs731236-rs7975232-rs1544410)	*VDR *	cs	**0.005**
rs4765623^∗^rs9679290	*SCARB1* ^∗^ *EPAS1 *	1.389 (1.094–1.763)	**0.007**
MC1R “r”^∗^rs7975232	*MC1R* ^∗^ *VDR *	1.455 (1.095–1.933)	**0.010**
rs7121^∗^rs2228570	*GNAS1* ^∗^ *VDR *	1.238 (1.009–1.518)	**0.041**

## References

[1] Storkel S., Eble J. N., Adlakha K. (1997). Classification of renal cell carcinoma: workgroup no. 1. Union Internationale Contre le Cancer (UICC) and the American Joint Committee on Cancer (AJCC). *Cancer*.

[2] Maubec E., Chaudru V., Mohamdi H. (2010). Characteristics of the coexistence of melanoma and renal cell carcinoma. *Cancer*.

[3] Yu M. C., Mack T. M., Hanisch R., Cicioni C., Henderson B. E. (1986). Cigarette smoking, obesity, diuretic use, and coffee consumption as risk factors for renal cell carcinoma. *Journal of the National Cancer Institute*.

[4] Chow W.-H., Gridley G., Fraumeni J. F., Järvholm B. (2000). Obesity, hypertension, and the risk of kidney cancer in men. *The New England Journal of Medicine*.

[5] Ferlay J., Steliarova-Foucher E., Lortet-Tieulent J. (2013). Cancer incidence and mortality patterns in Europe: estimates for 40 countries in 2012. *European Journal of Cancer*.

[6] Purdue M. P., Johansson M., Zelenika D. (2011). Genome-wide association study of renal cell carcinoma identifies two susceptibility loci on 2p21 and 11q13.3. *Nature Genetics*.

[7] Higgins J. P. T., Shinghal R., Gill H. (2003). Gene expression patterns in renal cell carcinoma assessed by complementary DNA microarray. *The American Journal of Pathology*.

[8] Han S. S., Yeager M., Moore L. E. (2012). The chromosome 2p21 region harbors a complex genetic architecture for association with risk for renal cell carcinoma. *Human Molecular Genetics*.

[9] Wu X., Scelo G., Purdue M. P. (2012). A genome-wide association study identifies a novel susceptibility locus for renal cell carcinoma on 12p11.23. *Human Molecular Genetics*.

[10] Henrion M., Frampton M., Scelo G. (2013). Common variation at 2q22.3 (*ZEB2*) influences the risk of renal cancer. *Human Molecular Genetics*.

[11] Purdue M. P., Ye Y., Wang Z. (2013). A genome-wide association study of renal cell carcinoma among African Americans. *Cancer Epidemiology Biomarkers and Prevention*.

[12] Frey U. H., Lümmen G., Jäger T. (2006). The *GNAS1* T393C polymorphism predicts survival in patients with clear cell renal cell carcinoma. *Clinical Cancer Research*.

[13] Karami S., Brennan P., Rosenberg P. S. (2009). Analysis of SNPs and haplotypes in vitamin D pathway genes and renal cancer risk. *PLoS ONE*.

[14] Bruyère F., Hovens C. M., Marson M.-N. (2010). VEGF polymorphisms are associated with an increasing risk of developing renal cell carcinoma. *The Journal of Urology*.

[15] Qin C., Cao Q., Li P. (2012). Functional promoter -31G>C variant in survivin gene is associated with risk and progression of renal cell cancer in a Chinese population. *PLoS ONE*.

[16] Wang W., Pan X., Huo X. (2012). A functional polymorphism C-1310G in the promoter region of Ku70/XRCC6 is associated with risk of renal cell carcinoma. *Molecular Carcinogenesis*.

[17] De Martino M., Haitel A., Schatzl G., Klingler H. C., Klatte T. (2013). The CASP8 -652 6N insertion/deletion promoter polymorphism is associated with renal cell carcinoma risk and metastasis. *The Journal of Urology*.

[18] Matsushita K., Takeuchi O., Standley D. M. (2009). Zc3h12a is an RNase essential for controlling immune responses by regulating mRNA decay. *Nature*.

[19] Mizgalska D., Wgrzyn P., Murzyn K. (2009). Interleukin-1-inducible MCPIP protein has structural and functional properties of RNase and participates in degradation of IL-1*β* mRNA. *The FEBS Journal*.

[20] Skalniak L., Mizgalska D., Zarebski A., Wyrzykowska P., Koj A., Jura J. (2009). Regulatory feedback loop between NF-*κ*B and MCP-1-induced protein 1 RNase. *The FEBS Journal*.

[21] Liang J., Saad Y., Lei T. (2010). MCP-induced protein 1 deubiquitinates TRAF proteins and negatively regulates JNK and NF-kappaB signaling. *Journal of Experimental Medicine*.

[22] Pośpiech E., Draus-Barini J., Kupiec T., Wojas-Pelc A., Branicki W. (2011). Gene-gene interactions contribute to eye colour variation in humans. *Journal of Human Genetics*.

[23] Pośpiech E., Wojas-Pelc A., Walsh S. (2014). The common occurrence of epistasis in the determination of human pigmentation and its impact on DNA-based pigmentation phenotype prediction. *Forensic Science International: Genetics*.

[24] Healy E., Jordan S. A., Budd P. S., Suffolk R., Rees J. L., Jackson I. J. (2001). Functional variation of MC1R alleles from red-haired individuals. *Human Molecular Genetics*.

[25] Nejentsev S., Godfrey L., Snook H. (2004). Comparative high-resolution analysis of linkage disequilibrium and tag single nucleotide polymorphisms between populations in the vitamin D receptor gene. *Human Molecular Genetics*.

[26] Dempfle A., Wudy S. A., Saar K. (2006). Evidence for involvement of the vitamin D receptor gene in idiopathic short stature via a genome-wide linkage study and subsequent association studies. *Human Molecular Genetics*.

[27] Sturm R. A., Duffy D. L., Box N. F. (2003). The role of melanocortin-1 receptor polymorphism in skin cancer risk phenotypes. *Pigment Cell Research*.

[28] Ritchie M. D., Hahn L. W., Roodi N. (2001). Multifactor-dimensionality reduction reveals high-order interactions among estrogen-metabolism genes in sporadic breast cancer. *The American Journal of Human Genetics*.

[29] Jakulin A., Bratko I. (2003). Analyzing attribute dependencies. *Knowledge Discovery in Databases: PKDD 2003*.

[30] Kontopantelis E., Reeves D. (2009). MetaEasy: a meta-analysis add-in for Microsoft Excel. *Journal of Statistical Software*.

[31] Altman D. G., Bland J. M. (2011). How to obtain the confidence interval from a P value. *British Medical Journal*.

[32] Williams D. L., Temel R. E., Connelly M. A. (2000). Roles of scavenger receptor BI and apo A-I in selective uptake of HDL cholesterol by adrenal cells. *Endocrine Research*.

[33] Shen W.-J., Hu J., Hu Z., Kraemer F. B., Azhar S. (2014). Scavenger receptor class B type I (SR-BI): a versatile receptor with multiple functions and actions. *Metabolism: Clinical and Experimental*.

[34] Danilo C., Gutierrez-Pajares J. L., Mainieri M. A., Mercier I., Lisanti M. P., Frank P. G. (2013). Scavenger receptor class B type I regulates cellular cholesterol metabolism and cell signaling associated with breast cancer development. *Breast Cancer Research*.

[35] Cao Q., Qin C., Ju X. (2012). Chromosome 11q13.3 variant modifies renal cell cancer risk in a Chinese population. *Mutagenesis*.

[36] Su T., Han Y., Yu Y. (2013). A GWAS-identified susceptibility locus on chromosome 11q13.3 and its putative molecular target for prediction of postoperative prognosis of human renal cell carcinoma. *Oncology Letters*.

[37] Colston K., Colston M. J., Feldman D. (1981). 1,25-dihydroxyvitamin D_3_ and malignant melanoma: the presence of receptors and inhibition of cell growth in culture. *Endocrinology*.

[38] Evans S. R. T., Houghton A. M., Schumaker L. (1996). Vitamin D receptor and growth inhibition by 1,25-dihydroxyvitamin D3 in human malignant melanoma cell lines. *The Journal of Surgical Research*.

[39] Okamoto T., Fujioka T., Horiuchi S. (1991). A study of the metabolism of vitamin D in patients with renal cell carcinoma. With special reference to serum concentration of 1*α*, 25-(OH)_2_D and its clinicla significance. *The Japanese Journal of Urology*.

[40] Fujioka T., Suzuki Y., Okamoto T., Mastushita N., Hasegawa M., Omori S. (2000). Prevention of renal cell carcinoma by active vitamin D3. *World Journal of Surgery*.

[41] Carlberg C., Seuter S., Heikkinen S. (2012). The first genome-wide view of vitamin D receptor locations and their mechanistic implications. *Anticancer Research*.

[42] Köstner K., Denzer N., Müller C. S. L., Klein R., Tilgen W., Reichrath J. (2009). The relevance of vitamin D Receptor (VDR) gene polymorphisms for cancer: a review of the literature. *Anticancer Research*.

[43] Bretherton-Watt D., Given-Wilson R., Mansi J. L., Thomas V., Carter N., Colston K. W. (2001). Vitamin D receptor gene polymorphisms are associated with breast cancer risk in a UK Caucasian population. *British Journal of Cancer*.

[44] Kim H. S., Newcomb P. A., Ulrich C. M. (2001). Vitamin D receptor polymorphism and the risk of colorectal adenomas: evidence of interaction with dietary vitamin D and calcium. *Cancer Epidemiology Biomarkers and Prevention*.

[45] Lurie G., Wilkens L. R., Thompson P. J. (2007). Vitamin D receptor gene polymorphisms and epithelial ovarian cancer risk. *Cancer Epidemiology Biomarkers and Prevention*.

[46] Kosiniak-Kamysz A., Marczakiewicz-Lustig A., Marcińska M. (2014). Increased risk of developing cutaneous malignant melanoma is associated with variation in pigmentation genes and VDR, and may involve epistatic effects. *Melanoma Research*.

[47] Toptaş B., Kafadar A. M., Cacina C. (2013). The vitamin D receptor (VDR) gene polymorphisms in Turkish brain cancer patients. *BioMed Research International*.

[48] Khan M. I., Bielecka Z. F., Najm M. Z. (2014). Vitamin D receptor gene polymorphisms in breast and renal cancer: current state and future approaches (Review). *International Journal of Oncology*.

[49] Gandini S., Gnagnarella P., Serrano D. (2014). Vitamin D receptor polymorphisms and cancer. *Advances in Experimental Medicine and Biology*.

[50] Ikuyama T., Hamasaki T., Inatomi H., Katoh T., Muratani T., Matsumoto T. (2002). Association of vitamin D receptor gene polymorphism with renal cell carcinoma in Japanese. *Endocrine Journal*.

[51] Obara W., Suzuki Y., Kato K., Tanji S., Konda R., Fujioka T. (2007). Vitamin D receptor gene polymorphisms are associated with increased risk and progression of renal cell carcinoma in a Japanese population. *International Journal of Urology*.

[52] Ou C., Zhao H. L., Zhu B., Huang L. S., Li P. Z., Lao M. (2014). Association of vitamin D receptor gene polymorphism with the risk of renal cell carcinoma: a meta-analysis. *Journal of Receptors and Signal Transduction*.

[53] Meng F., Ma P., Sui C. (2014). The association between VDR polymorphisms and renal cell carcinoma susceptibility: a meta-analysis. *Tumor Biology*.

[54] Morrison N. A., Qi J. C., Tokita A. (1994). Prediction of bone density from vitamin D receptor alleles. *Nature*.

[55] Moonesinghe R., Khoury M. J., Liu T., Ioannidis J. P. A. (2008). Required sample size and nonreplicability thresholds for heterogeneous genetic associations. *Proceedings of the National Academy of Sciences of the United States of America*.

[56] Moore J. H. (2003). The ubiquitous nature of epistasis in determining susceptibility to common human diseases. *Human Heredity*.

[57] Hahn L. W., Ritchie M. D., Moore J. H. (2003). Multifactor dimensionality reduction software for detecting gene-gene and gene-environment interactions. *Bioinformatics*.

[58] Lania A., Spada A. (2009). G-protein and signalling in pituitary tumours. *Hormone Research*.

[59] Frey U. H., Eisenhardt A., Lümmen G. (2005). The T393C polymorphism of the G*α*s gene (*GNAS1*) is a novel prognostic marker in bladder cancer. *Cancer Epidemiology Biomarkers and Prevention*.

[60] Arjumand W., Ahmad S. T., Nafees S. (2012). GNAS1 (G*α*s) gene T393C polymorphism and renal cell carcinoma risk in a north Indian population: a case-control study. *Genetic Testing and Molecular Biomarkers*.

[61] Kapellos G., Polonifi K., Farmakis D. (2013). Overexpression of Survivin levels in circulation and tissue samples of lung cancer patients. *Anticancer Research*.

[62] Song J., Su H., Zhou Y.-Y., Guo L.-L. (2013). Prognostic value of survivin expression in breast cancer patients: a meta-analysis. *Tumor Biology*.

[63] Lei Y., Geng Z., Guo-Jun W., He W., Jian-Lin Y. (2010). Prognostic significance of survivin expression in renal cell cancer and its correlation with radioresistance. *Molecular and Cellular Biochemistry*.

[64] Jang J. S., Kim K. M., Kang K. H. (2008). Polymorphisms in the survivin gene and the risk of lung cancer. *Lung Cancer*.

[65] Liu A. M. F., Lo R. K. H., Wong C. S. S., Morris C., Wise H., Wong Y. H. (2006). Activation of STAT3 by G*α*
_s_ distinctively requires protein kinase A, JNK, and phosphatidylinositol 3-kinase. *The Journal of Biological Chemistry*.

[66] Aoki Y., Feldman G. M., Tosato G. (2003). Inhibition of STAT3 signaling induces apoptosis and decreases survivin expression in primary effusion lymphoma. *Blood*.

[67] Yang W., Yoshigoe K., Qin X. (2014). Identification of genes and pathways involved in kidney renal clear cell carcinoma. *BMC Bioinformatics*.

[68] Miura S.-I., Fujino M., Matsuo Y. (2003). High density lipoprotein-induced angiogenesis requires the activation of Ras/MAP kinase in human coronary artery endothelial cells. *Arteriosclerosis, Thrombosis, and Vascular Biology*.

[69] Zamanian-Daryoush M., Lindner D., Tallant T. C. (2013). The cardioprotective protein apolipoprotein a1 promotes potent anti-tumorigenic effects. *The Journal of Biological Chemistry*.

[70] Haase V. H. (2009). The VHL tumor suppressor: master regulator of HIF. *Current Pharmaceutical Design*.

[71] Na X., Duan H. O., Messing E. M. (2003). Identification of the RNA polymerase II subunit hsRPB7 as a novel target of the von Hippel-Lindau protein. *The EMBO Journal*.

[72] Rini B. I., Small E. J. (2005). Biology and clinical development of vascular endothelial growth factor-targeted therapy in renal cell carcinoma. *Journal of Clinical Oncology*.

[73] Brudnik U., Branicki W., Wojas-Pelc A., Kanas P. (2009). The contribution of melanocortin 1 receptor gene polymorphisms and the agouti signalling protein gene 8818A>G polymorphism to cutaneous melanoma and basal cell carcinoma in a Polish population. *Experimental Dermatology*.

[74] Duffy D. L., Zhao Z. Z., Sturm R. A., Hayward N. K., Martin N. G., Montgomery G. W. (2010). Multiple pigmentation gene polymorphisms account for a substantial proportion of risk of cutaneous malignant melanoma. *The Journal of Investigative Dermatology*.

[75] Beisland C., Talleraas O., Bakke A., Norstein J. (2006). Multiple primary malignancies in patients with renal cell carcinoma: a national population-based cohort study. *BJU International*.

[76] Bradford P. T., Freedman D. M., Goldstein A. M., Tucker M. A. (2010). Increased risk of second primary cancers after a diagnosis of melanoma. *Archives of Dermatology*.

[77] Bertolotto C., Lesueur F., Giuliano S. (2011). A SUMOylation-defective MITF germline mutation predisposes to melanoma and renal carcinoma. *Nature*.

[78] Lindskog A., Ebefors K., Johansson M. E. (2010). Melanocortin 1 receptor agonists reduce proteinuria. *Journal of the American Society of Nephrology*.

[79] Hochberg Z., Templeton A. R. (2010). Evolutionary perspective in skin color, vitamin D and its receptor. *Hormones*.

