# Supplementary material for: Variants of SCARB1 and VDR Involved in Complex Genetic Interactions May Be Implicated in the Genetic Susceptibility to Clear Cell Renal Cell Carcinoma
Source: Biomed Res Int. 2015 Apr 6;2015:860405. doi: 10.1155/2015/860405 (PMC4402472; doi:10.1155/2015/860405)
Supplement: Supplementary file 1 — Supplementary Materials include four supplementary tables (PCR primer sequences for three multiplex reactions, Extension primer sequences for three multiplex reactions, Frequency of the haplotypes reconstructed with PHASE v2.1, Results of association logistic regression analysis between ccRCC status and single polymorphisms) and one supplementary figure (LD schemes generated with Haploview program for SNPs in EPAS1 (A), RXRA (B) and VDR (C) genes). [file 860405.f1.pdf]

	SNP-ID	Gene	Primer sequences	Concentration [μM]
Multiplex 1				
1	rs11894252	*EPAS1*	CATTGTGCTGTGTGTCTACCC	0.25
			CCCTAACCAGAGGCAGTCAG	0.25
2	rs7579899	*EPAS1*	TCTGTAGGGCATGACACAGC	0.125
			GCTGCTCTACCCAGGTGACT	0.125
3	rs3118523	*RXRA*	GGCAGTGGGGTTGATTACTG	0.125
			TCTGTCCTCACCTTCCCTGA	0.125
4	rs833061	*VEGFA*	TTCGAGAGTGAGGACGTGTG	0.1875
			AGGGAGCAGGAAAGTGAGGT	0.1875
5	rs748964	*RXRA*	CTTCCGTGGTCTGGAAAAGG	0.25
			CTCACTCTCACCCCAAGGAC	0.25
6	rs4765623	*SCARB1*	GCTCAGGGACATCACACGTA	0.125
			GATGCGTTCAGCAGTTCAGA	0.125
7	rs7105934	*11q13.3*	TCAGCTGAGGAATGATGAACA	0.25
			TTCCTGCCCCTACAGTCTTG	0.25
Multiplex 2				
1	rs132770	*XRCC6*	TAGAAGCTGGTTGGGGAGTG	0.25
			CTAGCTGGCGAACAACACAA	0.25
2	rs9904341	*BIRC5*	GCCATTAACCGCCAGATTTG	0.125
			GCAACGTCGGGGCACCCAT	0.125
3	rs9679290	*EPAS1*	TGTCCCTGAACTGAGGCTCT	0.125
			AAAGACCTTGCTTTGCCAGA	0.125
4	rs7121	*GNAS1*	CTGACCGCTTTGCTAAATCA	0.125
			CACAAGTCGGGGTGTAGCTT	0.125
5	rs4953346	*EPAS1*	AGGCATGGTTCTCTGCATAC	0.25
			AACCTCAGCGTCTATGAGTG	0.25
6	rs1049380	*ITPR2*	AAAAGGGTTTTGTTCCCTCTG	0.25
			CAAGCATTTTCTCATCTAAAAGTGT	0.25
Multiplex 3				
1	rs17849897	*ZC3H12A*	CCCTTACACGGGCTACAGTC	0.25
			GGGAAACACACCACACAGC	0.25
2	rs113322875	*ZC3H12A*	GCTGGGCTAGGAGCTAGGTT	0.125
			CAGGAAGTTTGAGGCTGGAA	0.125
3	rs34796867	*ZC3H12A*	AGCTGGTGAAACACGGGACA	0.0625
			GGGACTAGAGGGAGCTGAG	0.0625
4	rs113655247	*ZC3H12A*	CCCTTACACGGGCTACAGTC	0.0625
			GGGAAACACACCACACAGC	0.0625
5	rs34031609	*ZC3H12A*	TCATCCCAGTCCAGCTCTCT	0.125
			CTGATGGGGCATAGGTCTGT	0.125
6	rs12105918	*ZEB2*	CCACTGGAATTAGTACAACAAACAA	0.25
			AGGCAGGAGAATTGCTTGAA	0.25

Supplementary Table 1. PCR primer sequences for three multiplex reactions.

	SNP-ID	Neutral 5’→3’	Target 5’→3’	Size	Concentration [μM]
Multiplex 1					
1	rs11894252	----	AAAAAATACACACATTTCACT	21	1.20
2	rs7579899	----	GAACAGCTTGGTCGCAGTGCT	21	0.40
3	rs3118523	$(ct)_3$	CAAGCGGTCCAGTCAGGAGCC	27	0.70
4	rs833061	----	TGAGTGAGTGTGTGCGTGTGGGGTTGAGGG	30	0.60
5	rs748964	$t(ct)_{16}$	CAGCACTCAGCCATGTGCGCA	54	1.00
6	rs4765623	$(ct)_9$	CTCAGTAGATTTTGCCCAGTGGCTCTCC	46	0.20
7	rs7105934	$(ct)_{10}$	GCAGACTTTGAGTCGGCTTCTTAGCA	46	1.40
Multiplex 2					
1	rs132770	----	GCACATGCGTGATGACGTAG	20	0.40
2	rs9904341	$t(ct)_2$	ATTAACCGCCAGATTTGAATCGC	28	0.80
3	rs9679290	$(ct)_5$	CAGTGGCCTTGAGGTTTGTCAGAA	34	0.30
4	rs7121	$(ct)_{10}$	GAACCAGTTCAGAGTGGACTACAT	44	0.40
5	rs4953346	$(ct)_{13}$	CAGAGCCTCTGTGAGGAATAAATA	50	1.00
6	rs1049380	$(ct)_{13}$	TTATTTTAACTCAGAAAACATACT	50	1.00
Multiplex 3					
1	rs17849897	CAA	GCCTGCTCCTTGGCCAGGCTG	24	1.4
2	rs113322875	CTGACAA	CCCCGAGGTGGGTGCGT	24	0.15
3	rs34796867	AGTCTGACA A	GTGAGGTCTGGCGCTCCCGCT	31	0.5
4	rs113655247	GTCGTGAAA GTCTGACAA	GGCCAGCACCCATGGCCC	36	0.3
5	rs34031609	GTGAAAGTC TGACAA	AGCTTCTTCCCATCCAGGCTGCACT	40	0.1
6	rs12105918	----	CGTGAAAGTCTGACAATGCTATAAGTAACA TGCAGTTGTAAGCC	44	0.8

Supplementary Table 2. Extension primer sequences for three multiplex reactions.

Nr	Haplotype	Frequency [%]
**Chromosome 2, *EPAS1* gene:** rs11894252, rs7579899		
1	CC	64.20
2	CT	0.08
3	TC	0.17
4	TT	35.55
**Chromosome 2, *EPAS1* gene:** rs9679290, rs4953346		
1	GC	0.16
2	GA	54.49
3	CC	45.35
**Chromosome 2, *EPAS1* gene:** rs11894252, rs7579899, rs9679290, rs4953346		
1	CCGA	44.95
2	CCCC	19.21
3	CTGA	0.27
4	TCCC	0.14
5	TTGC	0.14
6	TTGA	9.54
7	TTCC	25.75
**Chromosome 9, *RXRA* gene:** rs748964, rs3118523		
1	GG	14.30
2	GA	0.14
3	CG	5.86
4	CA	79.70
**Chromosome 12, *VDR* gene, block 1: rs739837, rs731236, rs7975232, rs1544410**		
1	CAGC	51.63
2	CAGT	0.68
3	CGGC	0.27
4	CGTT	0.27
5	AAGC	0.41
6	AATC	11.04
7	AATT	0.82
8	AGGT	0.14
9	AGTC	0.54
10	AGTT	34.20
**Chromosome 12, *VDR* gene, block 3: rs2238136, rs4516035, rs7139166, rs11568820**		
1	GTCC	10.35
2	GTCT	11.58
3	GTGC	0.41
4	GTGT	0.41
5	GCCC	0.54
6	GCCT	0.27
7	GCGC	39.92

8	ATCC	30.93
9	ATCT	0.54
10	ACCC	0.41
11	ACGC	4.64

Supplementary Table 3. Frequency of the haplotypes reconstructed with PHASE v2.1.

Nr	Locus	Gene	Minor Allele	OR (95% CI)*	P value
1	rs34796867	*ZC3H12A*	T	monomorphic	-
2	rs113322875	*ZC3H12A*	A	monomorphic	-
3	rs34031609	*ZC3H12A*	A	monomorphic	-
4	rs113655247	*ZC3H12A*	C	monomorphic	-
5	rs17849897	*ZC3H12A*	T	0.793 (0.220-2.860)	0.724
6	rs12105918	*ZEB2*	G	1.198 (0.638-2.262)	0.579
7	rs11894252	*EPAS1*	T	1.142 (0.836-1.560)	0.405
8	rs7579899	*EPAS1*	T	1.185 (0.869-1.617)	0.284
9	rs9679290	*EPAS1*	C	1.259 (0.939-1.687)	0.124
10	rs4953346	*EPAS1*	C	1.247 (0.930-1.672)	0.140
11	rs3834129	*CASP8*	del	1.086 (0.974-1.445)	0.576
12	rs833061	*VEGFA*	C	1.056 (0.789-1.414)	0.714
13	rs748964	*RXRA*	G	0.907 (0.603-1.366)	0.643
14	rs3118523	*RXRA*	G	0.953 (0.660-1.380)	0.800
15	rs7105934	11q13.3	T	0.513 (0.227-1.158)	0.108
16	rs1049380	*ITPR2*	G	1.076 (0.806-1.434)	0.617
17	rs739837	*VDR*	A	0.929 (0.697-1.238)	0.614
18	rs731236	*VDR*	G	1.064 (0.791-1.431)	0.684
19	rs7975232	*VDR*	T	0.967 (0.724-1.291)	0.821
20	rs1544410	*VDR*	T	1.153 (0.861-1.544)	0.340
21	rs2228570	*VDR*	A	1.195 (0.884-1.617)	0.246
22	rs2238136	*VDR*	A	1.086 (0.786-1.501)	0.615
23	rs4516035	*VDR*	C	1.003 (0.741-1.355)	0.987
24	rs7139166	*VDR*	G	0.987 (0.731-1.335)	0.935
25	rs11568820	*VDR*	T	0.833 (0.539-1.286)	0.409
26	rs4765623	*SCARB1*	A	1.321 (0.971-1.798)<br>**1.688 (1.104-2.582)***	0.076<br>**0.016***
27	N29insA	*MC1R*	A	monomorphic	-
28	rs1805005	*MC1R*	T	1.079 (0.639-1.822)	0.776
29	rs1805006	*MC1R*	A	cs**	0.209
30	rs2228479	*MC1R*	A	1.062 (0.664-1.699)	0.801
31	rs11547464	*MC1R*	A	0.796 (0.131-4.821)	0.804
32	rs1805007	*MC1R*	T	0.973 (0.466-2.030)	0.941
33	Y152OCH	*MC1R*	A	monomorphic	-
34	rs1110400	*MC1R*	C	1.352 (0.536-3.409)	0.523
35	rs1805008	*MC1R*	T	1.318 (0.744-2.337)	0.344
36	rs885479	*MC1R*	T	1.434 (0.644-3.191)	0.378
37	rs1805009	*MC1R*	C	cs**	0.209
38	MC1R “R”	*MC1R*	“R”	1.227 (0.782-1.927)	0.373
39	MC1R “r”	*MC1R*	“r”	1.182 (0.843-1.657)	0.332
40	rs9904341	*BIRC5*	C	0.984 (0.727-1.332)	0.918
41	rs7121	*GNAS1*	T	1.244 (0.928-1.668)	0.144
42	rs132770	*XRCC6*	A	0.913 (0.599-1.392)	0.674

Supplementary Table 4. Results of association logistic regression analysis between ccRCC status and single polymorphisms. Significant results marked with bold.

* OR – the allelic odds ratio for the minor allele; CI - confidence interval

** cs – complete separation of the genotypes

*** dominant model

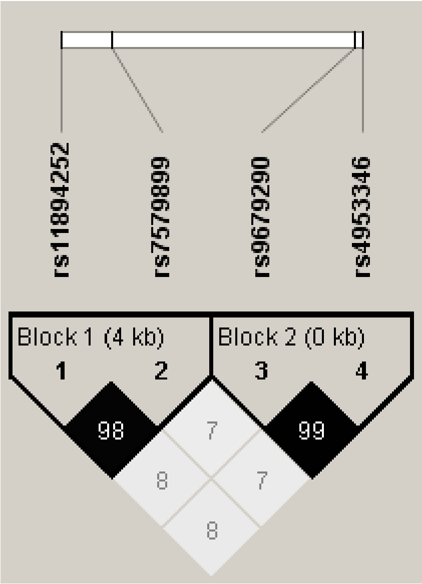
rs11894252
rs7579899
rs9679290
rs4953346
Block 1 (4 kb)
Block 2 (0 kb)
1
2
3
4
98
7
99
8
7
8
A

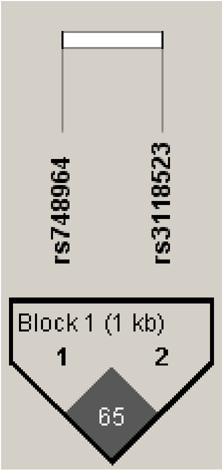
rs748964
rs3118523
Block 1 (1 kb)
1
2
65
B

C

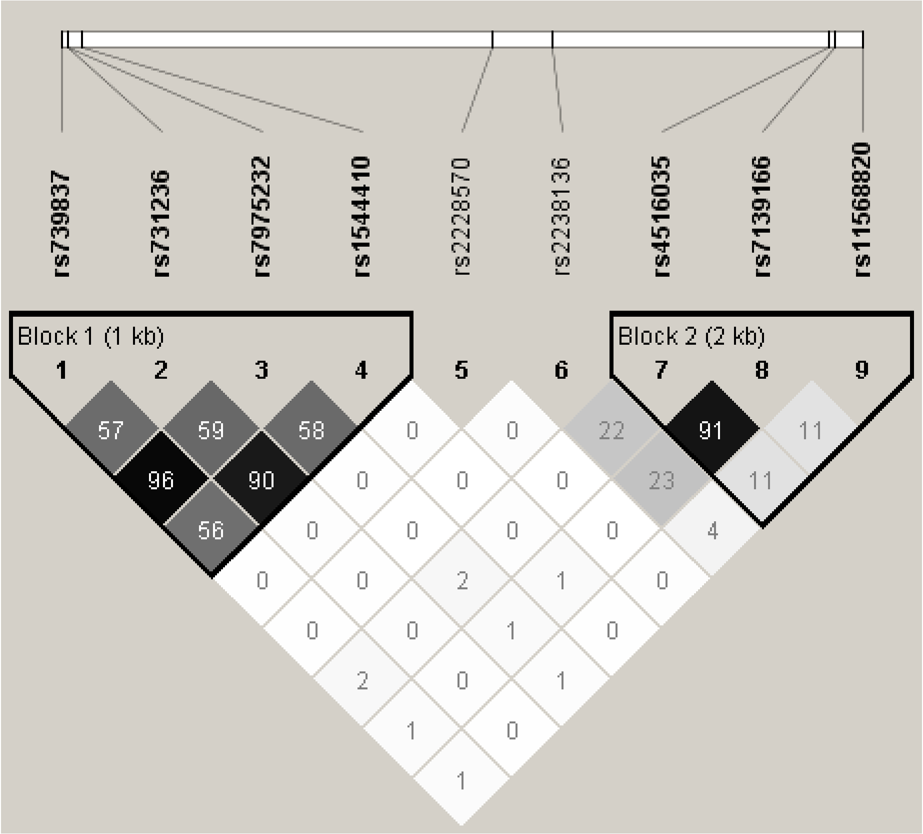

Supplementary Figure 1. LD schemes generated with Haploview program for SNPs in *EPAS1* (A), *RXRA* (B) and *VDR* (C) genes. Values of r^2 parameter are presented with white colour indicating r^2=0, shades of grey representing values of r^2 between 0-1 and black colour indicating complete LD with r^2=1. Values of r^2 are given in percentages.
